# Femoral Cancellisation Following Total Hip Arthroplasty With a Curved Triple Taper Polished Cemented Stem

**DOI:** 10.1016/j.artd.2025.101917

**Published:** 2025-12-05

**Authors:** Fumi Hirose, Tomohiro Yoshizawa, Shota Yasunaga, Koshiro Shimasaki, Ryunosuke Watanabe, Tomofumi Nishino, Hajime Mishima

**Affiliations:** Department of Orthopaedical Surgery, Institute of Medicine, University of Tsukuba, Tsukuba, Ibaraki, Japan

**Keywords:** Curved triple taper polished stem, Total hip arthroplasty, Cancellisation

## Abstract

**Background:**

Cancellisation is a radiolucent change occasionally observed at the bone–cement interface in cemented stems after total hip arthroplasty (THA). It is thought to reflect cortical bone remodeling due to osteoporosis or altered mechanical stress. This study aimed to investigate the characteristics, contributing factors, and mechanisms of cancellisation following THA using a curved triple-taper polished cemented stem.

**Methods:**

We retrospectively reviewed 61 hips who underwent primary THA with the curved triple-taper polished cemented stem between October 2012 and September 2019, with a minimum follow-up of 5 years. Cancellisation was assessed radiographically, and patients were grouped by its presence. Between-group comparisons included age, sex, body weight, femoral morphology, preoperative lumbar and femoral neck bone mineral density (BMD), bone turnover markers, stress shielding, changes in periprosthetic BMD, stem subsidence, and clinical outcomes score.

**Results:**

Cancellisation was found in 39 hips (64%), mainly in Gruen Zones 2, 3, and 6. No significant differences in age, sex, or body weight were observed between groups. The stovepipe femoral morphology was more frequent in the cancellisation group. The cancellisation group showed significantly lower BMD and elevated bone turnover marker. Stress shielding was more advanced and the bone density ratios around the stem were significantly reduced in zones 2, 3, 6, and 7. Moreover, there were no significant differences in stem subsidence or clinical outcome scores.

**Conclusions:**

Cancellisation is strongly associated with low systemic BMD and high bone turnover, suggesting a localized osteoporotic response. Its development may be influenced by altered load transmission from cemented stem fixation.

## Introduction

The SC stem (Kyocera, Kyoto, Japan) is a cemented curved triple-tapered polished femoral stem designed to enhance proximal load transfer and rotational stability compared to conventional triple-tapered polished stems. Its unique anterior–posterior curvature aims to achieve a more uniform stress distribution within the cement mantle, thereby potentially reducing the incidence of stress shielding [1].

**Figure 1:**
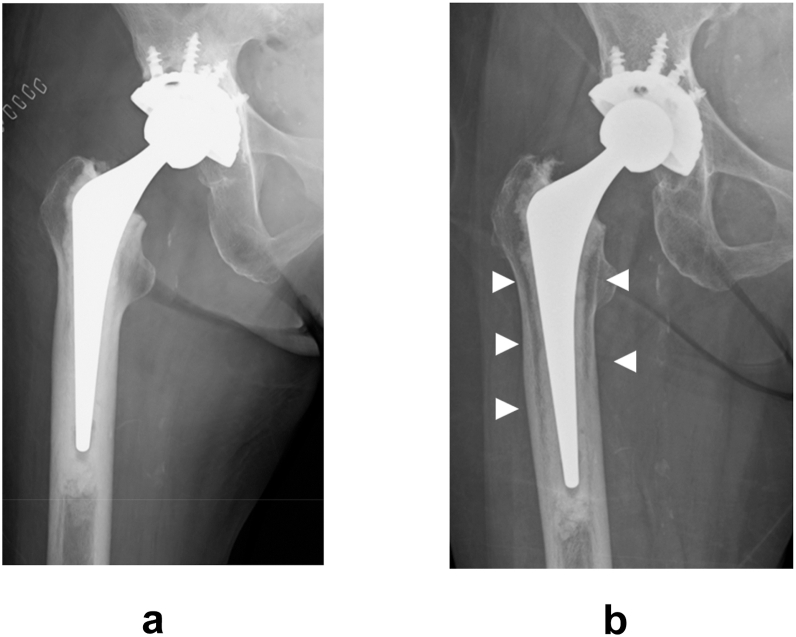
(a) Immediate postoperative plain radiographs, (b) plain radiographs at the final follow-up. The white triangular arrows indicate cancellisation.

However, during postoperative follow-up, a radiolucent change was occasionally observed at the bone–cement interface. Unlike the typical radiolucent line associated with loosening, this finding is characterized by a radiolucent change without accompanying sclerotic bone changes (Fig. 1). This radiolucent change was first histologically investigated by Kwong [2]. He reported that the change occurring at the bone–cement interface did not involve interposition of fibrous tissue, but rather consisted of cancellisation at the endosteal surface of the cortical bone and thinning of the trabeculae in the cancellous bone. Since then, this radiolucent change has often been collectively referred to as “cancellisation.” Cancellisation in cemented stems has been referred to as a manifestation of “cortical osteoporosis,” reflecting remodeling changes at the bone–cement interface. Previous radiographic and histological studies have described these remodeling patterns. Schmalzried et al. [3], in an anatomical study of well-fixed cemented stems, reported radiolucent change between the cement mantle and lateral cortex that represented a “secondary medullary canal” formed by radial trabecular bone. Similarly, Kwong et al. [2] and Jasty et al. [4] noted osteoporotic-like remodeling in the cortex without fibrous tissue interposition at the bone–cement interface but instead with trabecular bone formation in radial patterns. In Japanese clinical reports, its incidence has been reported to range from approximately 10%-50% [[5], [6], [7]]. In contrast, a radiolucent line indicative of fibrous tissue interposition typically presents with adjacent bone sclerosis and suggests loosening. Therefore, cancellisation is distinguished from radiolucent lines associated with implant failure. Although cancellisation has been interpreted as a manifestation of cortical bone osteoporotic changes or a remodeling response to altered mechanical stress, it is generally not considered a sign of prosthetic loosening [2]. However, the precise etiology and biological mechanisms underlying this phenomenon remain unclear.

This study aimed to elucidate the characteristics, contributing factors, and underlying mechanisms of cortical cancellous bone formation following total hip arthroplasty (THA) using SC stems at our institution.

## Material and methods

### Participants

**Figure 2:**
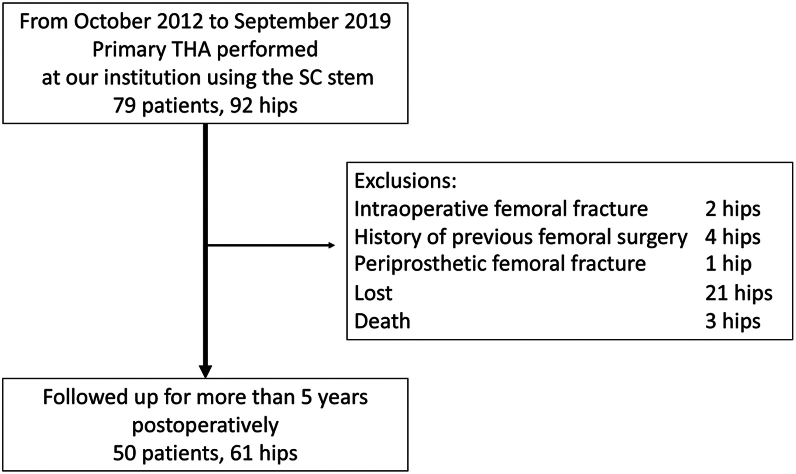
Flowchart of patient inclusion and exclusion.

This retrospective observational study included patients who underwent primary THA using an SC stem at the University of Tsukuba Hospital between October 2012 and September 2019. Among the initial 79 patients (92 hips), 50 (61 hips) with a minimum postoperative follow-up of 5 y were included in the analysis. The following cases were excluded: intraoperative femoral fractures (2 patients, 2 hips), prior femoral surgery (4 patients, 4 hips), periprosthetic femoral fractures within 5 years postoperatively (1 patient, 1 hip), patients lost to follow-up (20 patients, 21 hips), and deceased patients (3 patients, 3 hips) (Fig. 2).

Cementless stems were used for THA at our institution during the study period. The selection of cemented vs cementless stems was primarily based on patient age and femoral morphology during preoperative planning, reflecting individualized surgical decision-making. Cemented stems were chosen based on a comprehensive assessment, including elderly patients, cases with morphology close to stovepipe type or classified as Dorr type C femora, low bone mineral density (BMD), and underlying medical conditions.

This study was approved by the institutional review board of our hospital and was conducted in accordance with the relevant guidelines and regulations. Written informed consent was obtained from all participants before enrollment.

### Surgical procedures and postoperative management

All surgical procedures were performed using a posterior approach with the patient in the lateral decubitus position. Femoral cementation was performed using 80 g endurance bone cement (DePuy Synthes, Warsaw, IN) mixed with 800 mg amikacin. A third-generation cementing technique was used to ensure optimal fixation. For the distal centralization of the stem, a custom-made bone plug was created using cancellous bone harvested from the femoral head. The plug was inserted using an original impaction device equipped with a centralizing mechanism as previously described [8]. After thorough lavage of the femoral canal using a pulse lavage system for bone-bed preparation, the cement was vacuum-mixed and injected into a retrograde fashion using a cement gun. Proximal pressurization was performed with a pressurizer. The femoral stem was implanted at the depth at which its distal tip contacted the bone plug to achieve precise centralization within the canal.

Postoperative rehabilitation was initiated on postoperative day 1. The patients were allowed full weight-bearing ambulation as tolerated, along with both active and passive range of motion exercises and muscle strengthening of the operated hip. Discharge was typically scheduled for 2 to 3 weeks postoperatively, followed by outpatient follow-up.

Routine clinical and radiographic evaluations were conducted at 1 and 2 weeks, and 1, 3, 6, and 12 months postoperatively. Annual follow-up assessments were performed to monitor long-term outcomes and implant status.

### Assessments

Anteroposterior plain radiographs of the hip were used to evaluate the presence and distribution of cancellisation at the final follow-up. Distribution was assessed according to Gruen’s zone classification [9]. In this study, cancellisation was defined radiographically as a radiolucent change at the bone–cement interface without accompanying sclerotic bone changes, and radiographic evaluation was performed by comparing plain radiographs obtained immediately after surgery with those at the final follow-up. Based on this definition, each case was independently reviewed and categorized.

The following parameters were assessed for all patients:

1. Patient demographics: Age, sex, and body weight.
2. Femoral morphology: Cortical index [10] and canal flare index (CFI) [11]. Based on CFI values, femora were categorized as stovepipe (CFI <3.0) or champagneflute (CFI ≥4.7), and the proportions of each morphology were calculated.
3. Preoperative BMD was measured using dual-energy X-ray absorptiometry (DEXA; Lunar Prodigy, GE Healthcare, Chicago, IL) at the lumbar spine (L2–L4) and femoral neck on the operated side, expressed in g/cm.
4. Preoperative bone turnover markers (BTM) included tartrate-resistant acid phosphatase-5b (TRACP-5b) and total type I procollagen N-terminal propeptide (total P1NP).
5. Stress shielding was evaluated radiographically at the final follow-up using the Engh classification method [12], defined as follows: first degree: only the most proximal medial edge of the cut femoral neck was rounded off slightly; second degree: rounding off of the proximal medial femoral neck was combined with loss of medial cortical density at the femoral trochanter; third degree: more extensive resorption of cortical bone typically involved both the medial and anterior cortical regions at the femoral trochanter and the medial cortex at femoral subtrochanter; and fourth degree: cortical resorption extended below the femoral trochanter and subtrochanter into the diaphysis.
6. Periprosthetic BMD ratio: Quantified by DEXA at 1-week postoperative and final follow-up for each Gruen zone. The BMD ratio was calculated using the following formula:

BMD ratio (%) = (BMD at the final follow-up/BMD at 1 week postoperative) × 100.

7. Stem subsidence: The radiograph at the final follow-up was compared with the radiograph taken 1 week postoperative, and the measurement was performed using Fowler’s method [13].
8. The Japanese Orthopaedic Association (JOA) evaluation standard of hip joint function: The JOA score was evaluated at the final follow-up. The JOA Hip Score is a 100-point scale that comprises the subcategories of pain (40 points), range of movement (20 points), ability to walk (gait; 20 points), and activities of daily life (20 points) [14].

Radiographic assessments of stress shielding and cancellisation were independently interpreted by 3 hip surgeons with 18, 10, and 9 years of clinical experience, respectively.

### Statistical analysis

Participants were divided into 2 groups based on the presence or absence of cancellisation: the C group (with cancellisation) and the NC group (without cancellisation). Between-group comparisons of continuous variables were performed using the Mann–Whitney U test. The proportions of femoral morphology types (stovepipe and champagneflute) were compared between the groups using Fisher’s exact test.

Interobserver reliability for the radiographic interpretation of stress shielding and cancellisation was evaluated among the 3 hip surgeons. Fleiss’ kappa coefficient was used to assess the overall level of agreement among the observers. The degree of agreement was interpreted as follows: kappa coefficient = 0.00-0.20, slight; 0.21-0.40, fair; 0.41-0.60, moderate; 0.61-0.80, substantial; and 0.81-1.00, almost perfect agreement.

All statistical analyses were performed using IBM SPSS Statistics for Windows, Version 30.0.0.0 (Released 2024; IBM Corp., Armonk, NY). A 2-tailed *P* ≤ .05 was considered statistically significant.

## Results

### Patient demographics

The mean patient age at the time of surgery was 63.9 years (range, 36–84 years). The cohort consisted of 9 men (10 hips) and 41 women (51 hips). The mean follow-up duration was 7 years and 9 months (range, 5 years and 1 month to 10 years and 11 months).

The underlying diagnoses were as follows: osteoarthritis in 34, 11 hips with rheumatoid arthritis, 8 hips with osteonecrosis of the femoral head, 5 hips with rapidly destructive coxarthrosis, and 3 hips with femoral neck fracture.

### Presence and distribution of cancellisation

Cancellisation was observed in 39 hips, accounting for 64% of all the cases in this study. The distribution of cancellisation was most prominent in the lateral mid-to-distal femoral regions, with 35 hips (57%) showing involvement in Gruen Zone 2 and 31 hips (50%) in Gruen Zone 3. A relatively high frequency was also noted medially in Zone 6, with 22 hips (36%) exhibiting changes.

**Figure 3:**
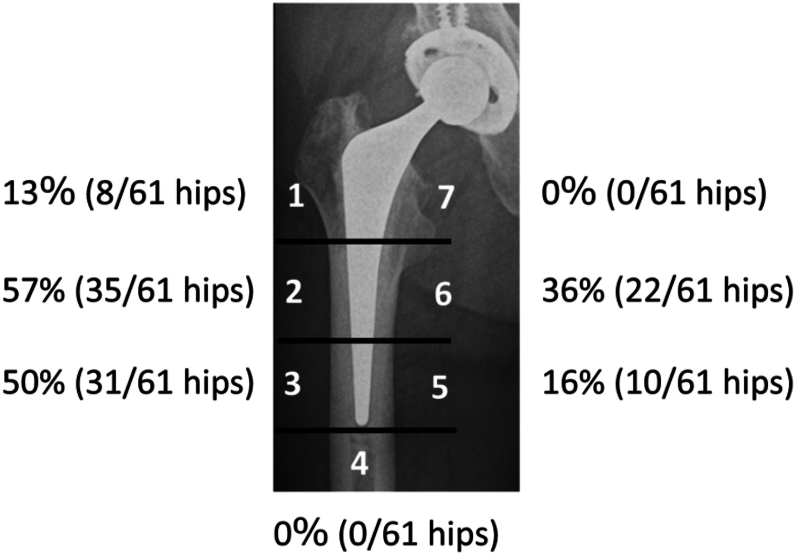
Distribution of cancellisation at the final observation.

By contrast, cancellisation was less frequently observed in Zones 1 (8 hips, 13%) and 5 (10 hips, 16%). No cases showed cancellisation in Zones 4 or 7 (Fig. 3). Cancellisation was observed in all 39 hips within 5 years postoperatively. In addition, 28 of the 39 hips (72%) showed evidence of cancellisation within 1 year after surgery. Among the 21 hips that were lost to follow-up within 5 years postoperatively, cancellisation was observed in 8 hips (38%).

### Comparison between the 2 groups

There were no significant differences in age, sex, or body weight between the C and NC groups (Table 1). However, group C tended to be older and had a lower body weight.

**Table 1:** Comparison of age, sex, and body weight between the C group and NC group.

Variables	C group	NC group	*P* value
Subjects (hips)	39	22	
Age at surgery (y)	65.74 ± 11.24	60.82 ± 11.97	.15
Sex (men/women)	7/32	3/19	.67
Body weight (kg)	51.49 ± 9.11	55.01 ± 13.95	.50

Values are in mean ± standard deviation.

Regarding femoral morphology, no significant differences were observed in the Cortical index between the groups. Nonetheless, the proportion of stovepipe-type femora was significantly higher in group C, with 16 hips (41%) exhibiting this morphology (Table 2).

**Table 2:** Comparison of femoral bone morphology between the C group and NC group.

Variables	C group	NC group	*P* value
CI	0.48 ± 0.06	0.48 ± 0.09	.79
Stovepipe (%)	41	18	.03^a^
Champagneflute (%)	11	22	.08

Values are in mean ± standard deviation.

CI, cortical index.

a *P* < .05.

The preoperative BMD was significantly lower in the C group for both the lumbar spine and femoral neck. Similarly, BTM, including TRACP-5b and total P1NP, were significantly higher in group C (Table 3).

**Table 3:** Comparison of BMD and BTM between the C group and NC group.

Variables	C group	NC group	*P* value
BMD (g/cm^2^)			
Lumber	0.80 ± 0.17	0.89 ± 0.17	.036^a^
Femoral	0.72 ± 0.19	0.87 ± 0.25	.005^b^
BTM			
TRACP-5b (mU/dL)	639.05 ± 367.34	392.55 ± 137.41	.0006^b^
Total P1NP (ng/ml)	76.06 ± 66.20	46.38 ± 23.54	.001^b^

Values are in mean ± standard deviation.

a *P* < .05.

b *P* < .01.

**Figure 4:**
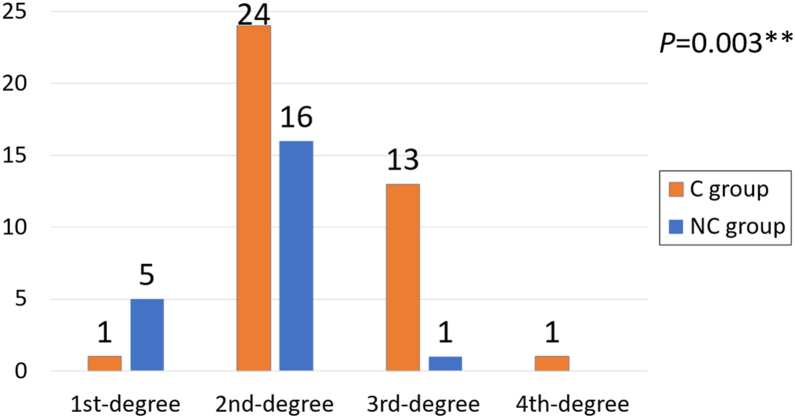
Comparison of the distribution of stress shielding at the final observation between the C group and the NC group. ∗∗*P* < .01.

A significant difference in the stress shielding severity was observed between the 2 groups. In group C, stress shielding was graded as follows: first degree in 1 hip, second degree in 24 hips, third degree in 13 hips, and fourth degree in 1 hip. In contrast, the NC group showed first degree in 5 hips, second degree in 16 hips, third degree in 1 hip, and no cases of fourth degree. These findings indicate more advanced stress shielding in Group C (Fig. 4).

The periprosthetic BMD ratios showed significant differences in zones 2, 3, and 6 where cancellisation was frequently observed. In the C group, the BMD ratios were reduced to 89.8% in zone 2, 99.9% in zone 3, and 89.4% in zone 6. In contrast, the NC group had ratios of 98.7%, 103.8%, and 98.4% in the same zones, respectively.

**Figure 5:**
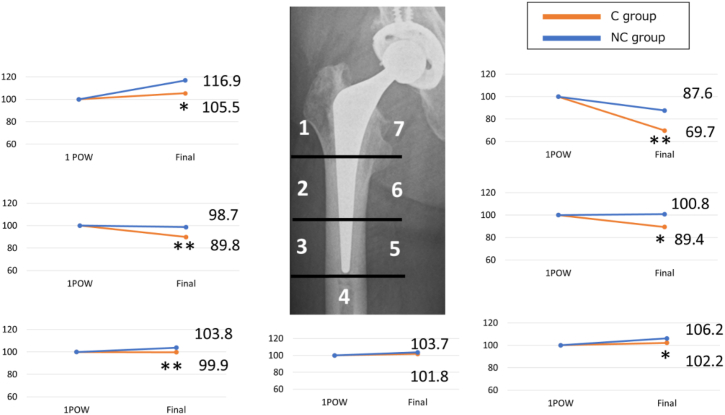
Comparison of the BMD ratio in each zone between 1 week postoperatively and the final observation between the C group and the NC group. ∗*P* < .05, ∗∗*P* < .01.

Although cancellisation was not observed in zone 7, the BMD ratio was significantly lower in group C (69.7%) than in group NC (87.6%). Additionally, significant differences in BMD ratios were noted in zones 1 and 5, although there was no evidence of a reduction in these zones. No significant changes were observed in zone 4 (Fig. 5).

The amount of stem subsidence was 0.86 mm in the C group and 0.82 mm in the NC group, with no significant difference between the groups (*P* = .15).

**Figure 6:**
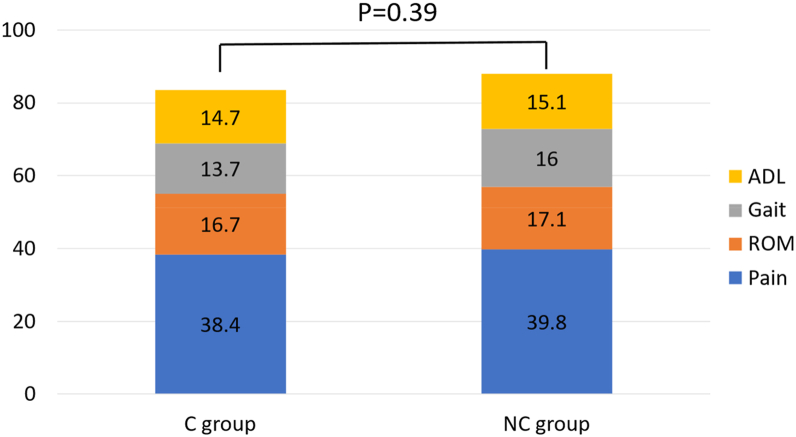
Comparison of JOA hip score between the C group and NC group. JOA, Japanese Orthopaedic Association; ADL, Activities of daily living; ROM, range of motion.

The total JOA hip score was 83.5 points in group C and 87.9 points in group NC, also showing no significant difference (Fig. 6). Moreover, there were no cases that required revision due to pain or loosening.

### Reproducibility of each interpreter

The interobserver reproducibility for the evaluation of stress shielding and cancellisation is summarized in Table 4. The level of agreement among the evaluators was classified as substantial for the assessment of stress shielding and almost perfect for the assessment of cancellisation, indicating the high reliability of radiographic interpretation for both parameters.

**Table 4:** Assessment of the reproducibility of each interpreter for each criterion.

Criterion	Total agreement Count	Agreement rate (%)	Kappa coefficient	95% confidence intervals
Stress shielding	48	79.00	0.70	0.59-0.81
Cancellisation	54	88.50	0.83	0.69-0.98

## Discussion

In this study, cortical cancellisation was observed in 64% of the hips that underwent THA using the SC stem. Cancellisation was most frequently located in Gruen Zones 2, 3, and 6, corresponding to the lateral mid-to-distal and medial midshaft regions of the femur. The group with cancellisation showed a significantly higher proportion of stovepipe-type femoral morphology, lower BMD, and elevated BTM. Furthermore, this group demonstrated more advanced stress shielding and significantly reduced periprosthetic BMD ratios in the areas where cancellisation was observed.

Previous studies have suggested that cancellisation is more common in elderly women and women with a stovepipe morphology, indicating a possible association with osteoporosis [5,15]. However, detailed evaluations of BMD and BTM have not been performed.

Our study not only confirmed the higher prevalence of stovepipe morphology in the cancellisation group (41% vs 18%) but also demonstrated significantly lower lumbar (0.80 vs 0.89 g/cm^2^, *P* = .036) and femoral neck BMD (0.72 vs 0.87 g/cm^2^, *P* = .005) and higher levels of both resorption and formation markers (TRACP-5b: 639.1 vs 392.6 mU/dL, *P* = .0006; P1NP: 76.1 vs 46.4 ng/ml, *P* = .001) , further supporting the association between cancellisation and underlying osteoporosis. The significantly elevated BTM in the cancellisation group suggested enhanced bone remodeling even before surgery, whereas the reduced BMD of the lumbar spine and femoral neck supported the presence of systemic osteoporosis preoperatively.

From a pathophysiological perspective, osteoporosis is characterized by increased bone remodeling that favors resorption, leading to cortical thinning and cancellisation of the endosteal surface of the cortical bone [16,17]. The term “cancellisation” reflects this process and aligns with the radiographic appearance observed in the present study. Stucinskas et al. [18] compared the aging changes in the operated and contralateral femora in patients with Müller cemented stems and found significantly greater cortical thinning on the operated side, indicating that implantation may accelerate bone loss beyond natural aging. These findings suggest that cancellisation represents the localized progression of osteoporosis in the femoral cortex following the implantation of a cemented stem.

Moreover, our study found that cases with cancellisation exhibited significantly more advanced stress shielding, with second to fourth degree stress shielding observed in 97% of cases, compared to only 44% in the NC group. BMD ratios in zones 2, 3, and 6—where cancellisation was most common—were significantly reduced in the C group (89.8%, 99.9%, and 89.4%, respectively) vs the NC group (98.7%, 103.8%, and 98.4%).

Stress shielding results from bone remodeling and are more commonly observed in patients with poor bone quality, including those with osteoporosis and stovepipe morphology [19]. Reduced BMD in stress shielding is a common characteristic with cancellisation. These results suggest that cancellisation and stress shielding may reflect sequential bone responses to altered mechanical environments in osteoporotic bone.

Despite similarities in etiology, cancellisation and stress shielding have distinct spatial and temporal patterns. Cancellisation tends to appear in zones 2 and 3, with a lateral distal predominance, as reported in studies using similar taper-polished stems, such as Exeter and CPT [5,15,20]. In contrast, stress shielding typically initiates proximally to the stem fixation site and progresses distally and from medial to lateral over time [12]. Stress shielding is classically associated with cementless stems owing to their rigid distal fixation of the stem and load transmission characteristics [12,19]. Given that cancellisation has consistently been reported in similar regions across taper-polished cemented stems (Exeter, CPT, SC stem), we believe that load transmission patterns specific to these cemented stem fixations may influence its occurrence.

Previous studies using thermoelastic stress analysis with femoral bone models have reported that compressive stress is applied to the proximal medial femur under loading, while tensile stress arises in the proximal lateral femur [21]. It has also been shown that insertion of a cementless stem alters this stress distribution within the femur [[21], [22], [23], [24]], and these stress changes are reported to correlate with postoperative variations in BMD [21]. In cementless stems that directly contact the femur, particularly taper-wedge stems, strong stress concentration has been observed in zone 2 [21]. In contrast, for cemented stems, we assume that the presence of cement interposed between the stem and femur likely reduces the stress transmitted to the bone. We assume that cancellisation—representing osteoporotic transformation of the cortical bone—occurs early at the sites where stress redistribution is greatest after cemented stem implantation, which may explain its frequent appearance in zones 2 and 3. Furthermore, as also suggested by our findings, this stress redistribution is presumed to be more pronounced in patients with osteoporosis. The development of cancellisation may be influenced by altered load transmission due to cemented stem fixation and by preexisting underlying osteoporosis. Future work is warranted to verify these mechanisms using thermoelastic stress analysis in femoral bone models.

Iwase et al. [20] found that heavier patients exhibited better preservation of the proximal femoral bone and a lower incidence of cancellisation when taper-polished cemented stems were used. Our findings also showed a trend toward lower body weight in patients with cancellisation. This supports the notion that hoop stress generated within the cement mantle due to stem “slipping” may stimulate bone formation in accordance with Wolff's law, particularly in heavier patients, thus preserving proximal BMD and preventing cancellisation. Conversely, in lighter patients, this mechanical stimulus may be insufficient, resulting in stress shielding, even with cemented stems. Taken together, our data suggests that cancellisation at the endosteal surface of the cortical bone may already occur preoperatively in patients with underlying osteoporosis. The implantation of a cemented stem may alter the local mechanical environment, triggering further remodeling, cortical thinning, and endosteal tabularization, ultimately producing the radiographic appearance of cancellisation. In this study, multivariate regression analysis demonstrated that body weight was a predictor of proximal femoral BMD preservation, with heavier patients maintaining BMD in zones 1 and 7, whereas lighter patients were more likely to experience bone loss. However, the report did not provide explicit body mass index thresholds or numerical definitions for “heavier” and “lighter” patients.

Previous reports have indicated that cancellisation is not associated with implant loosening or poor clinical outcomes [5]. There was no significant difference in stem subsidence between the C and NC groups (0.86 vs 0.82 mm, *P* = .15), and no cases required revision due to loosening. The absence of loosening or need for revision over a follow-up period exceeding 5 years supports the notion that cancellisation is not clinically related to implant loosening. Furthermore, clinical outcome scores, including pain-related measures, showed no significant differences (83.5 vs 87.9 points, *P* = .39), indicating no association between cancellisation and pain or functional outcomes.

This study has several limitations. First, although our results imply the influence of an altered load distribution, we did not perform mechanical or stress analyses. Future studies using surface stress mapping and finite element modeling may clarify the relationship between cancellisation and the mechanical environment. Second, BMD measurements around the stem may be affected by the inclusion of the cement mantle within the DEXA scan area, potentially leading to an overestimation of the true bone density. Third, although cancellisation was not associated with clinical outcome scores during follow-up periods exceeding 5 years and did not lead to loosening or the need for revision, it may potentially contribute to periprosthetic fractures or loosening due to other causes over longer-term follow-up of 15 to 20 years. Therefore, careful monitoring remains necessary. Fourth, although only one type of stem was evaluated in this study, this allowed for control of the stem-related variable, potentially enabling a more focused analysis of the associated risk factors and characteristics of cancellisation. Fifth, we did not consider pre- or postoperative osteoporosis treatment. Given the strong association between osteoporosis and cancellisation, further investigations are warranted to determine whether osteoporosis management can prevent or mitigate cancellisation. Finally, factors such as cement composition and polymerization heat were not evaluated. Sato [25] reported that even simple reaming of the femoral canal can cause cortical thinning and endosteal resorption and that cement filling may promote cancellisation, suggesting a direct influence of cement on bone remodeling.

## Conclusions

Cancellisation is significantly associated with low BMD and high BTM levels, indicating a strong relationship with osteoporosis. In addition, cancellisation represents a localized manifestation of osteoporotic changes in the femoral cortex. Furthermore, alterations in load transmission induced by cemented stems may contribute to the development of cancellisation.

## Conflicts of interest

The authors declare there are no conflicts of interest.

For full disclosure statements refer to https://doi.org/10.1016/j.artd.2025.101917.

## CRediT authorship contribution statement

**Fumi Hirose:** Writing – original draft, Visualization, Validation, Methodology, Investigation, Formal analysis, Data curation, Conceptualization. **Tomohiro Yoshizawa:** Writing – review & editing, Validation, Resources, Project administration, Methodology, Conceptualization. **Shota Yasunaga:** Validation. **Koshiro Shimasaki:** Formal analysis. **Ryunosuke Watanabe:** Writing – review & editing, Resources. **Tomofumi Nishino:** Writing – review & editing, Resources. **Hajime Mishima:** Supervision, Resources, Funding acquisition.

## Appendix A Supplementary data

Conflict of Interest Statement for Mishima

Conflict of Interest Statement for Watanabe

Conflict of Interest Statement for Nishino

Conflict of Interest Statement for Yoshizawa

Conflict of Interest Statement for Yasunaga

Conflict of Interest Statement for Shimasaki

Conflict of Interest Statement for Hirose
